# Blood Biomarkers for Evaluation of Perinatal Encephalopathy

**DOI:** 10.3389/fphar.2016.00196

**Published:** 2016-07-13

**Authors:** Ernest M. Graham, Irina Burd, Allen D. Everett, Frances J. Northington

**Affiliations:** ^1^Division of Maternal-Fetal Medicine, Department of Gynecology and Obstetrics, Johns Hopkins University School of MedicineBaltimore, MD, USA; ^2^Neuroscience Intensive Care Nursery Program, Johns Hopkins University School of MedicineBaltimore, MD, USA; ^3^Department of Neurology, Johns Hopkins University School of MedicineBaltimore, MD, USA; ^4^Integrated Research Center for Fetal Medicine, Johns Hopkins University School of MedicineBaltimore, MD, USA; ^5^Division of Cardiology, Department of Pediatrics, Johns Hopkins University School of MedicineBaltimore, MD, USA; ^6^Division of Neonatology, Department of Pediatrics, Johns Hopkins University School of MedicineBaltimore, MD, USA

**Keywords:** biomarkers, neonatal encephalopathy, hypoxic-ischemic encephalopathy, neuronal injury, Glial injury

## Abstract

Recent research in identification of brain injury after trauma shows many possible blood biomarkers that may help identify the fetus and neonate with encephalopathy. Traumatic brain injury shares many common features with perinatal hypoxic-ischemic encephalopathy. Trauma has a hypoxic component, and one of the 1st physiologic consequences of moderate-severe traumatic brain injury is apnea. Trauma and hypoxia-ischemia initiate an excitotoxic cascade and free radical injury followed by the inflammatory cascade, producing injury in neurons, glial cells and white matter. Increased excitatory amino acids, lipid peroxidation products, and alteration in microRNAs and inflammatory markers are common to both traumatic brain injury and perinatal encephalopathy. The blood-brain barrier is disrupted in both leading to egress of substances normally only found in the central nervous system. Brain exosomes may represent ideal biomarker containers, as RNA and protein transported within the vesicles are protected from enzymatic degradation. Evaluation of fetal or neonatal brain derived exosomes that cross the blood-brain barrier and circulate peripherally has been referred to as the “liquid brain biopsy.” A multiplex of serum biomarkers could improve upon the current imprecise methods of identifying fetal and neonatal brain injury such as fetal heart rate abnormalities, meconium, cord gases at delivery, and Apgar scores. Quantitative biomarker measurements of perinatal brain injury and recovery could lead to operative delivery only in the presence of significant fetal risk, triage to appropriate therapy after birth and measure the effectiveness of treatment.

## Introduction

Worldwide it is estimated that 1.15 million babies develop hypoxic-ischemic encephalopathy (HIE) every year (Lee et al., 2013). Up to 60% of infants with HIE will die or have severe disabilities by the age of 2 including mental retardation, epilepsy, and cerebral palsy (Pierrat et al., 2005). The costs related to HIE exceed $11 billion annually in the U.S. (Lawn et al., 2011). HIE is defined by a constellation of symptoms in the neonate, and no definitive diagnostic test is available (American College of Obstetricians Gynecologists, 2014). One of the greatest challenges in perinatal medicine is assessing the fetus during labor and the neonate shortly after birth for evidence of brain injury. The presence of meconium, non-reassuring fetal heart rate tracing, Apgar scores, umbilical artery blood gases, and physical exam, are tools currently used to identify brain injury in the fetus and neonate but they all both separately and collectively lack precision. Amplitude integrated EEG can detect early changes associated with brain injury and has been used to determine prognosis and predict long term outcomes, (Merchant and Azzopardi, 2015) however, interference from hypothermic environments can reduce the prediction of HIE prognosis, and amplitude integrated EEG cannot determine the time of injury (Thoresen et al., 2010).

One of the major challenges that needs to be solved is the early discrimination of mild-moderate injury from severe injury. The availability of therapies such as whole-body hypothermia, which must be instituted within 6 h of birth, make the rapid identification of a baby with neurologic injury critically important. How to objectively and quantitatively identify the fetus and neonate with brain injury may be solved by borrowing an approach from traumatic brain injury research. Extensive effort has been applied in the field of traumatic brain injury to identify acute blood biomarkers as diagnostics to identify these patients, discriminate severity, monitor treatment efficacy and as prognostics for recovery and long-term disabilities (Diaz-Arrastia et al., 2014).

Blood biomarkers are utilized as a diagnostic tool to identify patients with a possible disease or abnormal condition, e.g., elevated glucose levels for the diagnosis of diabetes mellitus and elevated levels of cardiac troponin for diagnosis of acute myocardial infarction (Atkinson et al., 2001). Biomarkers can be used to predict the stage of a disease and its severity, such as measuring the concentration of prostate-specific antigen in the blood to detect the level of tumor growth and metastasis (Mouhieddine et al., 2015). Following brain injury a destructive cascade of biological events continues over hours and days that may worsen the patient's condition following a primary insult and a secondary reperfusion phase. The focus of biomarker development in traumatic brain injury has been on the identification of moderate to severe injury; (Mondello et al., 2012a) however, the greatest potential impact of blood biomarkers to change clinical practice in perinatology is for mild injury since diagnostic and prognostic challenges presented for mild injuries are more difficult to identify and monitor (Diaz-Arrastia et al., 2014). There is no current standard therapy for mild injury and increasingly late outcomes suggest that mild injury results in identifiable pathology.

Similar to traumatic brain injury, it is unlikely that a single biomarker will reflect the full picture of the injured brain for a multifaceted complex disease such as HIE (Diaz-Arrastia et al., 2014). Combining biomarkers from multiple cellular pathways has shown superior sensitivity and specificity in the identification of traumatic brain injury (Diaz-Arrastia et al., 2014). Simultaneous measurements of neuronal and glial biomarkers may complement each other to identify distinct injury mechanisms and determine the timing of injury. A study of severe traumatic brain injury found that glial biomarker elevations are primarily a reflection of focal mass lesions, and that diffuse injuries primarily result in neuronal biomarker elevations (Mondello et al., 2012a). Since patients with diffuse injuries may require different therapies from those with focal lesions, such a combination of biomarkers may enable us to select patients for targeted therapies (Saatman et al., 2008; Diaz-Arrastia et al., 2014). In perinatal medicine this might allow differentiation of large major vessel stroke from more diffuse hypoxic-ischemic injuries. The ideal biomarker panel may include multiple biomarkers produced by different brain cell types, and currently available multiplex immunoassay platforms make it possible to measure up to 10 biomarkers with a high degree of sensitivity from small volumes of plasma (Diaz-Arrastia et al., 2014). Investigators have proposed developing a point of care handheld device that can quickly and accurately measure brain injury biomarkers similar to the handheld dextrometer of diabetic patients that can measure a glucose level within seconds (Bressan et al., 2014).

Optimally, the level of the brain injury biomarkers should correlate with the size, location and severity of the lesion, clinical outcome and response to treatment (Mouhieddine et al., 2015). Ideally, serum biomarkers should provide information on the pathophysiology of injury, improve stratification of patients by injury severity, assist in the monitoring of secondary insults and injury progression, monitor response to treatment and predict functional outcome (Papa et al., 2008). Circulating brain injury biomarker levels in neonatal HIE could indicate brain injury and reflect the extent of damage, solving a clinical dilemma in the discrimination of mild vs. moderate-severe injury (Lv et al., 2015).

**Figure 1:**
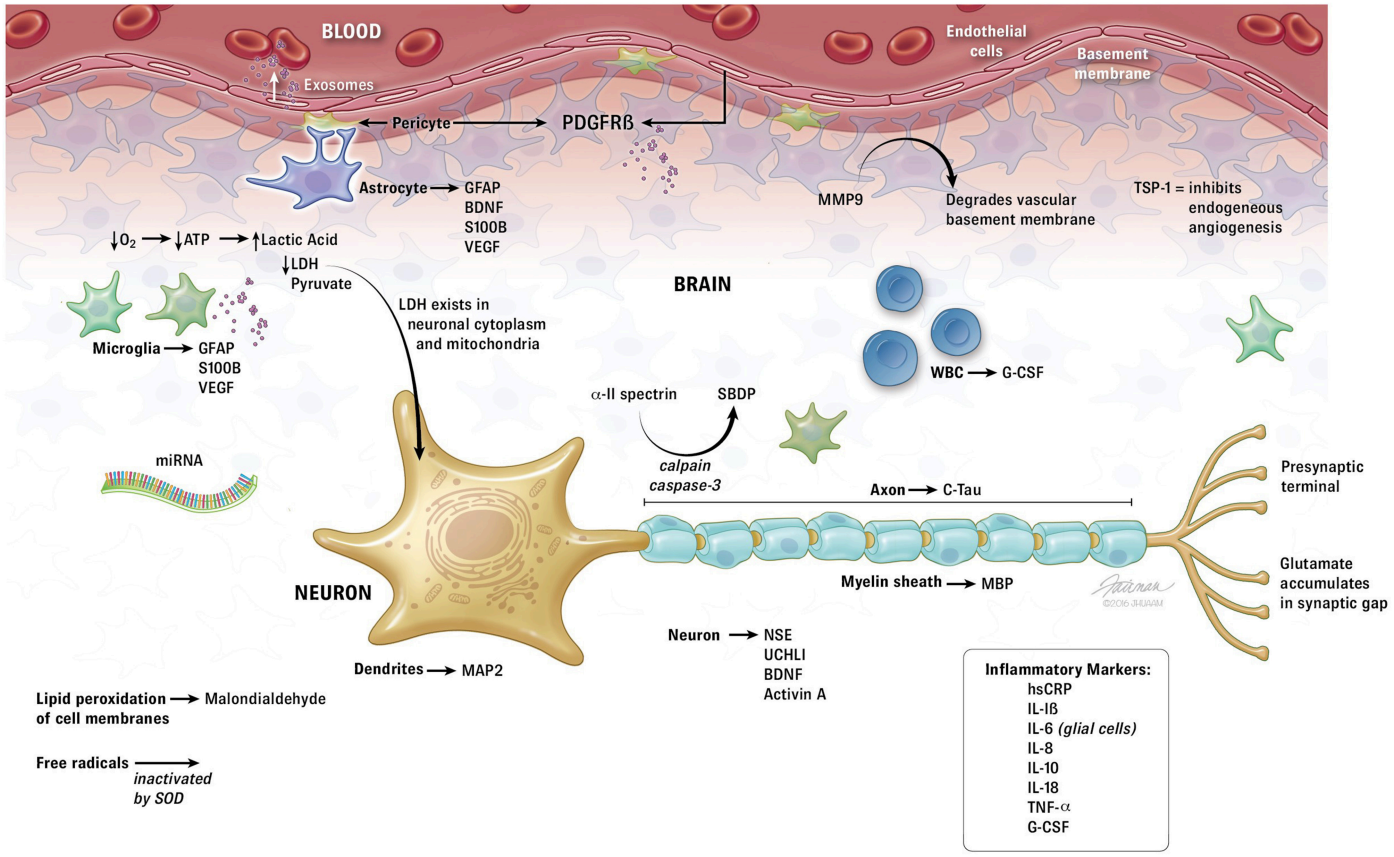
**Summary of biomarkers related to perinatal hypoxic-ischemic encephalopathy**. ATP, adenosine triphosphate; BDNF, brain derived neurotrophic factor; C-Tau, cleaved-Tau; G-CSF, granulocyte colony stimulating factor; GFAP, glial fibrillary acidic protein; hsCRP, high sensitivity C-reactive protein; IL, interleukin; LDH, lactate dehydrogenase; MAP, microtubule-associated protein; MBP, myelin basic protein; miRNA, micro ribonucleic acid; MMP, matrix metalloproteinase; NSE, neuron specific enolase; PDGFR, platelet derived growth factor receptor; SBDP, spectrin breakdown products; SOD, superoxide dismutase; TNF, tumor necrosis factor; TSP, thrombospondin; UCH, ubiquitin carboxy-terminal hydrolase; VEGF, vascular endothelial growth factor.

Brain injury biomarker protein discovery can be either hypothesis driven or discovery driven. In the discovery driven method samples from normal and brain injured patients are compared with mass spectrometry to identify differences in circulating brain proteins. Unlike hypothesis driven research that identifies potential biomarkers before testing them in cases and controls, discovery driven research collects a huge amount of information first then extracts questions and answers from the data (Guingab-Cagmat et al., 2013). As currently there is no gold standard for diagnosing mild traumatic brain injury, not even by conventional assessment through neuroimaging techniques, (Niogi and Mukherjee, 2010) these approaches have been applied to identify brain specific markers of injury. During brain injury, neural proteins or their breakdown products are released into the extracellular environment reaching the cerebrospinal fluid (CSF) in relatively high concentration, and the blood stream via the compromised blood-brain barrier (Guingab-Cagmat et al., 2013; Figure 1). In premature infants in particular, the blood brain barrier is particularly fragile, potentially increasing the likelihood that brain proteins may reach the circulation after injury (Dammann and Leviton, 1997). Clearance and half-life of the biomarkers contribute to the final concentration that can be measured in blood (Guingab-Cagmat et al., 2013). Review of recent research in traumatic brain injury suggests significant potential for crossover of traumatic brain injury biomarkers to the brain injured fetus and neonate. The following biomarkers and classes of biomarkers may be used as tools to diagnose neonatal brain injury, follow its treatment efficacy and provide prognostic information.

## Glial fibrillary acidic protein (GFAP)

Of the numerous candidate biomarkers for traumatic brain injury GFAP holds the most promise (Guingab-Cagmat et al., 2013). One of the main strengths of GFAP as a brain injury biomarker is that it is only found within the central nervous system (CNS; Galea et al., 1995). GFAP is a cytoskeletal intermediate filament protein that forms networks that support astroglial cells and is found only in the astroglial cytoskeleton (Guingab-Cagmat et al., 2013). Astrocytes are important to brain injury because their foot processes comprise part of the blood brain barrier, and with disruption after injury astrocyte damage results in early release of GFAP (Vos et al., 2004; Guingab-Cagmat et al., 2013). GFAP levels peak at 1–2 days following severe brain trauma, and are normal in patients with other trauma that does not include traumatic brain injury, an indication of GFAP's brain specificity (Schiff et al., 2012). In a study of 81 patients with traumatic brain injury, serum GFAP levels in the 1st 24 h post-injury were significantly higher in patients with a focal mass lesion compared to patients with diffuse injury (Mondello et al., 2011). GFAP may be an appropriate and sensitive candidate for the diagnosis of focal brain injury such as contusion or intracerebral hemorrhage (Yokobori et al., 2013). GFAP may also be important in the differential diagnosis of various types of stroke, which is clinically relevant as immediate treatment of stroke depends on whether the stroke is ischemic or hemorrhagic (Guingab-Cagmat et al., 2013). In neonates, serum GFAP levels have been shown to be significantly elevated at the time of birth and during the first week of life in term and near-term infants with HIE that have abnormal brain MRI scans at 1 week of life (Ennen et al., 2011; Massaro et al., 2013; Chalak et al., 2014) and in premature neonates that develop periventricular white matter injury (Stewart et al., 2013). In addition, GFAP may provide insights into the pathobiology of therapeutic hypothermia as significant elevations in GFAP occur after rewarming from therapeutic cooling for HIE in the neonates that later have an abnormal MRI, suggesting that the neonates with severe injury manifest reperfusion injury post-therapuetic cooling (Schiff et al., 2012). This may offer an opportunity to discriminate mild from moderate to severe HIE and triage high risk neonates to evolving adjunctive therapies.

## Neuron-specific enolase (NSE)

NSE is 1 of 5 isozymes of the glycolytic enzyme enolase found in central and peripheral neurons and red blood cells and has been shown to be elevated following cell injury (Skogseid et al., 1992). NSE is not normally secreted into extracellular fluids by intact neurons, but when axons are damaged NSE is upregulated in an attempt to maintain homeostasis (Yokobori et al., 2013). It is highly expressed in neuronal cytoplasm and has been shown to have the sensitivity and specificity to detect neuronal cell death (Selakovic et al., 2005). Given its location and abundance NSE should possess relatively high specificity and sensitivity for axonal injury, and accordingly NSE seems to have excellent potential as a therapeutic monitor in the neurological intensive care unit and for the determination of long-term prognosis (Gradisek et al., 2012). A limitation of NSE is its lack of brain specificity, being abundant in red blood cells which leads to the occurrence of false positive results in the setting of hemolysis (Papa et al., 2008). Increased cerebrospinal fluid (CSF) and serum levels of NSE have been reported after traumatic brain injury, and NSE concentrations were associated with severity of injury, CT scan abnormalities and outcome (Selakovic et al., 2005; Guingab-Cagmat et al., 2013). NSE has been shown to provide quantitative measures of brain damage and to improve the diagnosis and prediction of ischemic stroke, intracerebral hemorrhage, seizures, comatose patients after cardiopulmonary resuscitation for cardiac arrest, and traumatic brain injury (Isgro et al., 2015). There is a positive correlation between NSE levels and infarct volume after acute ischemic stroke, and serum levels of NSE in the first few days of ischemic stroke may serve as a useful marker to predict stroke severity and early functional outcome (Isgro et al., 2015).

Release patterns of NSE and S100B were investigated in 66 traumatic brain injury patients, and in those with cortical contusions NSE was highest on the 1st measurement on day 1 after injury, decreasing from this peak over 4 days, while S100B slowly increased over the 4 day period (Herrmann et al., 2000). Serum concentrations of NSE and S100B were significantly correlated with contusion volume, but the 1st sample taken was the most valid indicator of traumatic brain injury severity (Herrmann et al., 2000). Similarly in 152 pediatric traumatic brain injury patients higher NSE concentrations were associated with worse outcome, and initial and peak NSE concentrations correlated with Glasgow Outcome Scale score at all time points investigated, particularly in children ≤ 4 years of age (Berger et al., 2007).

## S100B

S100B is the principal low affinity calcium-binding protein in astrocytes and is considered a marker of astrocyte injury and death (Papa et al., 2008). Although S100B is glial specific and expressed primarily by astrocytes and Schwann cells, it is also found in several non-nervous system cells such as adipocytes, chondrocytes, skin, glioblastoma, and melanoma cells (Zimmer et al., 1995). S100B has a serum half-life of only 2 h, (Guingab-Cagmat et al., 2013) but is stable in blood and not affected by hemolysis (Lv et al., 2015). Since S100B is produced outside the CNS, general trauma without brain injury can increase its levels (Rothoerl and Woertgen, 2001). S100B spikes up after hemorrhagic shock and correlates with shock severity which decreases it's value as a single biomarker for traumatic brain injury (Guingab-Cagmat et al., 2013). It may be possible to use S100B as a biomarker of brain injury if measured immediately after injury; however, most mild brain injury patients are not evaluated at the time the injury occurs (Guingab-Cagmat et al., 2013).

S100B levels in blood at 24 h post-traumatic brain injury provides an early and sensitive biomarker for the prediction of brain damage which may be useful in defining early risk patterns (Egea-Guerrero et al., 2013). Elevated serum levels of S100B correlate with MRI abnormality and neuropsychological examination after mild traumatic brain injury (Ingebrigtsen et al., 1999). In 265 patients with traumatic brain injury S100B levels added substantial information regarding patient outcome in excess of that provided by age, Glasgow Coma Scale, pupil reaction and CT scan, and elevated S100B levels were best correlated to CT-visible intracranial pathology (Thelin et al., 2013). It has been estimated that adding the measurement of S100B to clinical decision tools for mild traumatic brain injury patients could potentially reduce the number of CT scans by 30% (Biberthaler et al., 2006). Furthermore, S100B below a threshold level may also safely eliminate the need to obtain a CT scan in patients with mild traumatic brain injury (Unden et al., 2013). A significant correlation has been found between the volume of contusion visible on CT scan and serum S100B concentration (Raabe et al., 1998).

Cord blood concentration of S100B > 2.20 μg/L has a sensitivity of 87% and specificity of 88% in predicting moderate to severe HIE (Qian et al., 2009). Serum concentrations of S100B 2 h after birth of 8.5 μg/L predict the occurrence of severe neonatal HIE (Nagdyman et al., 2001). Salivary S100B levels may also be able to predict neonatal neurologic abnormalities related to asphyxia (Gazzolo et al., 2015). In patients with severe traumatic brain injury S100B appears to be a more accurate biomarker for predicting intracranial hypertension than NSE, particularly in the early post-injury phase (Yokobori et al., 2013). In neonates with HIE treated with hypothermia or normothermia, both NSE and S100B levels were highly elevated following asphyxia, however, serum S100B levels were lower in the hypothermia group and strongly correlated with the neurodevelopmental outcome (Roka et al., 2012).

## Ubiquitin carboxy-terminal hydrolase L1 protein (UCHL1)

UCHL1, also known as neuronal-specific protein gene product 9.5, is a cysteine protease that is predominately expressed in neurons, but is also expressed in small amounts in neuroendocrine cells (Guingab-Cagmat et al., 2013). This small (25 kDa) enzyme comprises about 2% of the total soluble protein in the brain and hydrolyzes the C-terminal bond of ubiquitin or unfolded polypeptides (Setsuie and Wada, 2007). Because of its high and specific expression in brain tissue, increased levels of UCHL1 have been suggested as a marker of CNS injury such as acute cerebral ischemic disease and early stage severe traumatic brain injury (Lv et al., 2015). Serum UCHL1 is a useful biomarker of severe traumatic brain injury both in the acute phase and in the 1st week after injury (Mondello et al., 2012b). In 251 patients with suspected mild to moderate traumatic brain injury UCHL1 outperformed GFAP and S100B in reducing CT scan use without sacrificing sensitivity (Welch et al., 2016). Of the 3 biomarkers, UCHL1 had the best test performance to differentiate between subjects with normal vs. abnormal CT scans (Welch et al., 2016). When obtained within 6 h of injury UCHL1 in combination with GFAP was very sensitive for a positive head CT, and was thought to provide the objective evidence clinicians desire when trying to reduce the use of CT scans in patients with mild traumatic brain injury (Welch et al., 2016). Serum UCHL1 levels were higher in patients with diffuse injury, in contrast to GFAP levels which were higher in patients with focal mass lesions (Mondello et al., 2011). High levels of UCHL1 have been found in the umbilical cord blood of neonates with HIE associated cortical injury and subsequent movement and cognitive disorders (Massaro et al., 2013; Douglas-Escobar et al., 2014).

## Cleaved Tau (C-Tau)

Tau is an intracellular, microtubule associated protein that is highly enriched in axons which stably assembles axonal microtubule bundles and participates in anterograde axoplasmic transport (Yokobori et al., 2013). Tau is preferentially localized in the axon, and Tau lesions are related to axonal disruption (Higuchi et al., 2002). Under normal conditions axonal Tau is below the level of detection, however, after axonal injury activated calpain depolymerizes microtubules in both perikarya and neurites to form filamentous Tau inclusions which are a pathologic hallmark of axonal injury (Yokobori et al., 2013). A cleaved form of Tau has been investigated as a potential biomarker of CNS injury (Papa et al., 2008). C-Tau levels in the CSF are significantly elevated after traumatic brain injury, and these levels correlate with clinical outcome (Zemlan et al., 2002). Although levels of C-Tau were also elevated in plasma from patients with severe traumatic brain injury, there was no correlation between plasma levels and clinical outcome (Chatfield et al., 2002). Serum Tau protein concentrations are significantly increased in bilirubin encephalopathy of newborns, and there is significant correlation between Tau levels and the severity of brain injury (Okumus et al., 2008).

## Microtubule-associated protein 2 (MAP2)

MAP2 is primarily expressed in the nervous system and is one of the most abundant proteins in the brain (Yokobori et al., 2013). MAP2 is generally dendrite-specific and potentially a good candidate biomarker for dendritic injury (Kobeissy et al., 2008). A study of 16 patients with severe traumatic brain injury found that serum MAP2 concentrations correlated with neurologic outcome at 6 months after injury (Mondello et al., 2012c). Perinatal asphyxia has been shown to affect the distribution of MAP2 in the brainstem of children (Covenas et al., 2014).

## Myelin basic protein (MBP)

MBP is the major component of the myelin sheath, and has a crucial role in the maintenance of myelin structure and function (Barbarese et al., 1988). It is one of the most abundant proteins in white matter, representing 30% of the protein content of myelin (Yokobori et al., 2013). Under normal conditions only a small amount is released into the blood stream, but in white matter brain injury the concentration of MBP in blood and CSF increases rapidly reflecting the severity of myelin damage which allows MBP to be used as a specific biomarker of white matter lesions or nerve fiber demyelination (Lv et al., 2015). MBP diminishes significantly in the contused rat cortex as early as 2 h after traumatic brain injury reaching its lowest level at 48 h (Ottens et al., 2008). Serum and CSF MBP has been studied as a biomarker for traumatic brain injury and for determination of outcome (Yokobori et al., 2013). Levels of serum MBP in neonates with moderate to severe HIE was significantly higher than those with mild HIE and no injury (Lv et al., 2015).

## Spectrin breakdown products (SBDP)

Alpha-II-spectrin is the main structural component of the cortical membrane cytoskeleton and is particularly abundant in axons and presynaptic terminals (Riederer et al., 1986). Alpha-II-spectrin is a major substrate for both calpain and caspase-3 cysteine proteases, and is cleaved into breakdown products that may serve as biomarkers of brain injury (Guingab-Cagmat et al., 2013). A signature of caspase-3 and calpain activation is cleavage of several common proteins such as cytoskeletal alpha-II-spectrin (Ringger et al., 2004). Since calpain and caspase-3 are major executioners of necrotic and apoptotic cell death during ischemia and trauma, SBDP may provide crucial information not only on the severity of brain injury, but also on the underlying pathophysiological mechanisms associated with cell death (Guingab-Cagmat et al., 2013). Calpain and caspase-3 mediated SBDP levels in CSF are significantly increased in patients after traumatic brain injury (Pineda et al., 2007). Average SBDP values measured early after injury correlate with severity of injury, CT scan findings and outcome at 6 months post-injury (Pineda et al., 2007). This is consistent with activation of a continuum of cell death mechanisms over different time courses following severe traumatic brain injury, and shows that SBDP are potentially useful biomarkers of severe traumatic brain injury and may provide information about the timing of injury (Papa et al., 2008).

## Brain-derived neurotrophic factor (BDNF)

BDNF is a neurotrophin secreted by CNS neurons and astrocytes that is involved in neuronal survival and synaptic plasticity. BDNF promotes the growth, differentiation, regeneration and repair of neurons (Lv et al., 2015). Proinflammtory cytokines enhance neurotrophic signaling via expression of BDNF (Werner and Stevens, 2015). Decreased levels of serum BDNF have been associated with higher clinical severity of traumatic brain injury and with decreased 6-month functional outcome scores (Korley et al., 2016). After traumatic brain injury serum BDNF is acutely decreased correlating with injury severity, and therapies that increase brain BDNF expression, such as environmental enrichment, show promise for cognitive recovery (Failla et al., 2016). In 113 traumatic brain injury patients with serum BDNF levels measured 0–6 days (acute) and 6–12 months (chronic) post-injury, serum BDNF levels were reduced after traumatic brain injury at all time points (Failla et al., 2016). Acute serum BDNF may be a viable predictive biomarker for mood and cognitive complications within the 1st year after traumatic brain injury, and BDNF may provide a treatment window in the acute phase that affects long term recovery (Failla et al., 2016). Chronic serum BDNF may be reflective of injury severity and serve as a potential biomarker for tracking treatment response and effectiveness in real-time (Failla et al., 2016). If serum BDNF levels are persistently elevated in neonates with HIE this suggests severe brain injury and a poor prognosis (Imam et al., 2009).

## Activin A

Activin A is a trophic factor that regulates neuron proliferation and is a member of the transforming growth factor β superfamily (Lv et al., 2015). Activin A has been shown to protect from hypoxic-ischemic damage in cell culture and animal models (Mukerji et al., 2007). Hydrogen peroxide treatment increased activin mRNA twofold in surviving cortical neurons, and inhibition of activin with neutralizing antibodies caused neuronal death (Mukerji et al., 2007). After transient focal cerebral ischemia in adult mice, activin mRNA increased at 1 and 4 h ipsilateral to the infarct but returned to control values at 24 h after reperfusion (Mukerji et al., 2007). Activin was also increased after 2 h of 11% hypoxia (Mukerji et al., 2007). Activin mRNA increased at 1 h, but not 4 or 24 h after hypoxia, similar to the time course of erythropoietin and vascular endothelial growth factor induction (Mukerji et al., 2007). This shows that activin is an early-regulated gene response to transient ischemia and hypoxia (Mukerji et al., 2007). Because activin responds to oxidative challenge protecting neurons, it may have a role as a potential therapy in stroke injury (Mukerji et al., 2007). In full term neonates with moderate to severe HIE, activin A is significantly elevated in CSF, and it may be a reliable early indicator for the identification of HIE (Imam et al., 2009).

## Matrix metalloproteinase-9 (MMP-9)

MMP-9 is involved in the breakdown of the blood-brain barrier by degrading brain vasculature basement membrane components (Lv et al., 2015). Under the effect of inflammatory mediators and oxygen free radicals MMP-9 is activated, and the basement membrane of the blood-brain barrier is damaged, increasing permeability and causing secondary vascular source cerebral edema, which is part of the pathophysiology of HIE (Lv et al., 2015). Serum MMP-9 is significantly elevated in neonates with HIE, and its elevation is related to the time of onset (Liu et al., 2009). A sustained increase of serum MMP-9 concentrations in neonates with HIE indicates worsening blood-brain barrier damage leading to brain damage and edema (Lv et al., 2015).

## Vascular endothelial growth factor (VEGF)

VEGF is an angiogenic factor secreted by astrocytes and microglia that is overexpressed in hypoxia-ischemia and protects neurons and glial cells by promoting the proliferation and angiogenesis of vascular endothelial cells (Lv et al., 2015). Brain VEGF mRNA expression increases and reaches a sustained peak at 12 h of life for a duration of 14 days or longer in animal models of hypoxia-ischemia (Liang and Wang, 2005). Plasma concentrations of VEGF increase with increased severity of neonatal HIE (Lv et al., 2015).

## Platelet derived growth factor receptor β (PDGFRβ)

The integrity of the blood-brain barrier is essential for proper neuronal functioning, and pericytes are crucial for maintaining blood-brain barrier integrity. Brain capillary pericyte dysfunction results in blood-brain barrier breakdown and contributes to neurological injury (Sagare et al., 2015). PDGFRβ is expressed in the brain by vascular mural cells, brain capillary pericytes and arterial vascular smooth muscle cells, and is a marker for blood-brain barrier disruption (Sagare et al., 2015). In cultures of human brain pericytes exposed to hypoxia PDGFRβ is a biomarker of pericyte injury (Sagare et al., 2015). Elevated PDGFRβ in biofluids in patients with neurodegenerative disorders likely reflects ongoing pericyte injury and supports its potential to be developed and validated as a biomarker of brain pericyte injury and blood-brain barrier dysfunction (Sagare et al., 2015). Inflammation, induced either by trauma or hypoxia, can induce blood-brain barrier disruption by altering tight junction function leading to paracellular leakage and affecting vesicular processes leading to transcytotic leakage of potential biomarkers (Banks et al., 2015). To date PDGFRβ has not been studied in neonatal HIE.

## Thrombospondin-1 (TSP-1)

Angiogenesis is a fundamental endogenous process for brain development and repair. TSP-1 is the 1st identified endogenous angiogenesis inhibitor, and its expression is upregulated after intracerebral hemorrhage (Dong et al., 2015). TSP-1 may be released into the CSF from damaged brain tissue with recirculation into the peripheral blood (Dong et al., 2015). In 110 patients with intracranial hemorrhage compared to age and gender matched healthy controls increased plasma TSP-1 concentrations following intracranial hemorrhage were independently associated with injury severity and short and long term clinical outcomes (Dong et al., 2015). TSP-1 may be a useful complementary tool to acutely assess the severity of injury and predict poor clinical outcomes following acute intracranial hemorrhage which may make it an ideal biomarker to determine prognosis in premature neonates with intraventricular hemorrhage. TSP-1 has not been studied in neonates with HIE.

## Inflammation related markers

Because neuroinflammation is such a prominent feature of traumatic brain injury, cytokines have been investigated as potential biomarkers. Cytokines play both harmful and curative roles in traumatic brain injury, readily cross the blood-brain barrier and may relay important information on the severity and prognosis of injury (Banks et al., 2016). High sensitivity C-reactive protein is a sensitive marker of inflammation and tissue injury whose concentration increases rapidly in brain tissue following hypoxia-ischemia. In 74 neonates with HIE high sensitivity C-reactive protein, interleukin-6 and tumor necrosis factor-α (TNF-α) were significantly increased, and high levels correlated with a poor prognosis (Shang et al., 2014). High sensitivity C-reactive protein reaches a peak at day 3 of life then begins to decrease; if the serum concentration fails to decrease the prognosis is poor (Tian and Yan, 2012).

Interleukin-1β (IL-1β) promotes brain damage through the release of free radicals, stimulating inflammatory reactions, and enhancing the toxicity of excitatory amino acids (Lv et al., 2015). In neonates with HIE elevated serum IL-1β levels are associated with neurological abnormalities at 6–12 months (Liu and Feng, 2010). In 16 deceased full term asphyxiated infants there was increased expression of IL-1β in the hippocampus in those with seizures, and all cases with seizures displayed alteration in the blood-brain barrier as assessed by immunohistochemistry for albumin (Schiering et al., 2014). The authors speculated that seizure development may lead to secondary brain damage, and that IL-1β may aid in the development of therapeutic targets for neonatal seizures (Schiering et al., 2014).

IL-6 is produced by glial cells and has a protective effect on the CNS by inhibiting the synthesis of TNF-α and IL-1 and promoting nerve growth factor secretion. However, high concentrations of IL-6 can induce inflammation and increase vascular permeability leading to cerebral edema (Lv et al., 2015). When the cord blood of 50 neonates with HIE was compared to 113 controls, IL-6 levels were significantly elevated, aiding the diagnosis of brain injury, and were related to prognosis (Wu et al., 2012).

IL-8 is a neutrophil chemotaxis factor that recruits neutrophils to injured areas, and through enhanced IL-1β and TNF-α neurotoxicity increases brain injury (Lv et al., 2015). IL-8 increases in the acute phase of injury in neonates with HIE, and the more severe the injury the higher the IL-8 levels (Lv et al., 2015). In a study of 13 neonates with HIE and epilepsy, most inflammatory factors in the serum were decreased after 8–72 h, however, serum IL-8 levels remained high indicating that IL-8 might be an early biomarker for the diagnosis of neonatal HIE with epilepsy (Youn et al., 2012).

IL-10 plays a protective role in brain tissue by inhibiting the secretion of IL-1β, IL-8, and TNF-α, inhibiting the production of chemokines, decreasing leukocyte aggregation, and reducing inflammatory responses in the brain (Lv et al., 2015). IL-10 levels are significantly elevated in the acute phase of injury in neonates with HIE (Wang et al., 2003).

IL-18 is an anti-inflammatory factor that stimulates the expression of IL-1β and IL-8, and can both protect brain tissue and aggravate brain damage (Felderhoff-Mueser et al., 2005). Serum levels of IL-18 are elevated in neonates with HIE, and levels correlate with the severity of brain damage (Guo et al., 2014).

## Tumor necrosis factor alpha (TNF-α)

Systemic inflammation leads to increased CNS inflammation and injury through direct transport of inflammatory agents or inflammatory cells across the blood-brain barrier, and it has been proposed that infection-induced upregulation of TNF-α can produce or worsen brain injury (Leviton, 1993). This hypothesis is supported by various studies showing that pro-inflammatory cytokines such as TNF-α produced by the placenta can cross the blood-brain barrier (Jin et al., 2015).

Intra-amniotic infections lead to preterm delivery which increases the risk of neurologic morbidity in preterm neonates. Elevated amniotic fluid levels of the inflammatory cytokines TNF-α, IL-1β, and IL-6 are linked with white matter injury in preterm neonates, (Yoon et al., 1997) and elevated cord blood levels of these cytokines correlate with neonatal cerebral lesions on MRI (Duggan et al., 2001). In preterm infants, an elevated inflammatory response during the perinatal period correlates with long term morbidities including cerebral palsy, necrotizing enterocolitis, bronchopulmonary dysplasia, and chronic lung disease (Jin et al., 2015). Elevated inflammatory cytokines measured in neonatal blood correlate with periventricular white matter injury, ventriculomegaly and severe germinal matrix hemorrhage assessed by ultrasound (Nelson et al., 2003). At this point extensive literature strongly supports the bioplausibility of all of these inflammatory cytokines as biomarkers of neonatal HIE.

## Granulocyte colony stimulating factor (G-CSF)

G-CSF mobilizes stem cells and is currently used to promote the production of neutrophils in chemotherapy patients that develop neutropenia. G-CSF has neuroprotective properties after peripheral administration and is protective against carbon monoxide toxicity and ischemia (Banks et al., 2016). Serum levels of G-CSF correlate with severity of stroke (Yu et al., 2012) and are diagnostic for gliomas (Yildiz et al., 2011). G-CSF was the only cytokine of 23 measured whose serum levels were elevated after traumatic brain injury in a murine model (Dohi et al., 2014). High levels of G-CSF are associated with better functional outcome and reduced lesion volume in intracranial hemorrhage (Sobrino et al., 2009). G-CSF is elevated 2 h after controlled cortical injury, but not at later time points in mice, which may make it useful in determining the timing of injury (Dohi et al., 2014). Plasma G-CSF levels correlate with neuroinflammation in the same mouse models (Dohi et al., 2014) and in human studies where they correlate with time since injury and total severity of injury (Banks et al., 2016). In a neonatal rat HIE model treatment with G-CSF has been shown to attenuate long term brain damage (Fathali et al., 2010).

## Oxidative stress related markers

Free radicals cause lipid peroxidation of cell membranes, and the antioxidant enzyme superoxide dismutase and the lipid peroxidation product malondialdehyde reflect the extent of oxidative damage to cells (Lv et al., 2015). Excess free radicals consume a large amount of superoxide dismutase and produce a large amount of malondialdehyde, which may allow these compounds to be used for the early prediction of neonatal HIE, but they are not brain specific (Qin et al., 2005).

## Metabolism related markers

Since perinatal hypoxia-ischemia leads to brain injury through an increase in anaerobic metabolism, clinical testing of metabolites may help identify the neonate with HIE and follow their recovery (Lv et al., 2015). Hypoxia leads to an increase in anaerobic glycolysis, decreased ATP production, and accumulation of lactic acid. Lactate dehydrogenase exists in neuronal cytoplasm and mitochondria, and its role is to catalyze the oxidation of lactate to pyruvate. At the onset of neonatal HIE lactate dehydrogenase activity and lactate production are increased, and a combination of lactate dehydrogenase and NSE may be used to identify brain injury (Lv et al., 2015). Detection of lactate dehydrogenase with other metabolites may have an important role in neonatal HIE diagnosis and prognostic evaluation (Lv et al., 2015).

Extracellular glutamate primarily mediates excitotoxicity and is involved in the pathophysiological processes of brain ischemia (Lv et al., 2015). During HIE ATP synthesis decreases, glutamate transport is inhibited and glutamate accumulates in the neuronal synaptic cleft subsequently leading to neuronal death (Lv et al., 2015). Because glutamate is brain specific, it can be a sensitive biomarker for brain injury, and if monitored early may aid in the early diagnosis of HIE in the neonate and could be a marker of therapeutic efficacy as ancillary therapies are developed for HIE (Lv et al., 2015).

## MicroRNA (miRNA)

MicroRNA (miRNA) are being investigated as promising serum biomarkers for neurotrauma. miRNA may be stable in the circulation which supports their potential use as disease biomarkers (Egea et al., 2012). miRNAs are endogenously expressed ~22 nucleotide long noncoding RNAs that control a wide spectrum of cellular function, and bind to target regions of certain genes to control their expression by either repression or activation of mRNA translation/transcription (Chen et al., 2015). miRNAs play important roles in developmental and functional aspects of the CNS and in many neurological diseases, which potentially make them important candidates for brain injury diagnosis (Chen et al., 2015). Emerging data suggest that exosomal miRNA may provide potential biomarkers in acute ischemic stroke (Chen et al., 2015). Studies of endothelial cell cultures and *in vivo* rat focal ischemia models have shown significant reductions in serum miR-126 detected at 3 h after permanent ischemia but not transient ischemia, which suggests that changes in serum miR-126 may be able to distinguish severe permanent ischemia from milder injury after transient ischemia (Chen et al., 2015). miR-126 was selected as the initial candidate biomarker of neurovascular damage in stroke because it is specifically and highly expressed in endothelial cells and is known to be involved in the regulation of vascular integrity, endothelial function, and angiogenesis (Chen et al., 2015). miRNAs have been shown to be altered in plasma before the first spontaneous seizure and have been proposed as putative biomarkers of epileptogenesis (Roncon et al., 2015). Recently miRNAs have been found to be involved in the pathophysiology of HIE, including the regulation of excitatory amino acid toxicity, oxidative stress, inflammatory reactions and apoptosis (Lv et al., 2015). Plasma miRNA profiles compared between severe traumatic brain injury patients and healthy volunteers found that decreases in the levels of miR-16 and miR-92a and increased levels of miR-765 were good markers of severe traumatic brain injury at 25–48 h after injury (Redell et al., 2010). Specific miRNAs seem to be good candidate biomarkers for distinguishing focal and diffuse brain injury or for accurate determination of raised intracranial pressure (Yokobori et al., 2013). Animal models of neonatal HIE have shown specific miRNA changes following injury using microarray (Weiss et al., 2012). Expression of miR-21 is found in astrocytes. A study of 49 cases of neonatal HIE showed that serum miR-21 was significantly increased and may be a marker for the early diagnosis of neonatal HIE (Chen and Yang, 2012).

## Exosomes

A potentially transformative finding in traumatic brain injury research has been the identification of exosomes, which are nano-sized extracellular vesicles that have key roles in cell signaling and undergo membrane fusion so that they readily cross the blood-brain barrier. Exosomes contain proteins, miRNA and other nucleic acids, and are believed to play a major role in disposal of cellular waste (Werner and Stevens, 2015). Exosomes may serve as vehicles for targeted delivery of repair-inducing molecules and possibly as novel biomarkers (Werner and Stevens, 2015). They have been implicated in an array of signaling processes involving astrocytes, oligodendrocytes, microglia, neurons, and neural stem cells (Rajendran et al., 2014). Increased levels of exosomes have been found after traumatic brain injury in humans (Patz et al., 2013). Injured neurons release the microtubule-associated protein Tau which is then carried by exosomes (Rajendran et al., 2014). Exosomes may represent ideal biomarker containers, as RNA and protein transported within the vesicles are protected from enzymatic degradation, and there is considerable interest in developing assays to evaluate blood-borne brain-derived exosomes, often referred to as the “liquid brain biopsy” (Werner and Stevens, 2015).

## Conclusion

Blood biomarkers discovered in traumatic brain injury could significantly improve the management of neonates with HIE, particularly those with mild and moderate injury, by providing more accurate early diagnosis and prognosis, and for monitoring therapies in the acute care setting (Papa et al., 2008). Biomarkers could help determine severity and mechanism of injury and quantitatively measure injury progression which would provide major opportunities for clinical research (Papa et al., 2008).

Serum based combined multi-marker analysis reflecting glial and neuronal cell damage should be considered for understanding more precise pathophysiological mechanisms of brain injury. Glial injury can be assessed by GFAP in the blood and axonal injury by C-Tau and spectrin protein breakdown products (Guingab-Cagmat et al., 2013). The serum levels of glial and neuronal biomarkers (S100B, GFAP, UCHL1) at the time of admission after brain injury have been shown to correlate with clinical outcome and are sensitive and specific in determining the severity of injury (Lee et al., 2015). With UCHL1 levels increasing in diffuse injury and GFAP levels increasing in focal injury, a glial:neuronal ratio has been proposed as a novel indicator to differentiate focal and diffuse injury, and has been found to be more accurate when measured at < 12 h after injury (Mondello et al., 2012a). Several potential epilepsy biomarkers have been proposed in recent years including blood biomarkers of inflammation, blood-brain barrier damage and brain injury. Given the complexity of epilepsy it is unlikely that a single biomarker is sufficient for predicting epileptogenesis, but a combinatorial approach may be able to identify appropriate biomarkers at different stages of the evolution of the disease (Loscher et al., 2013).

The pathology of perinatal HIE is very heterogeneous and one “magic” biomarker may not be the solution, but a panel of biomarkers may prove to be most useful in distinguishing the different pathologic-anatomic processes that comprise the injury (Papa et al., 2008). The brain consists of many elements, and depending on the mechanism and severity of injury, various damage patterns may be reflected by different combinations of biomarkers (Lee et al., 2015). Biomarkers will probably supplement existing tools, such as the Glascow Coma Scale and neuroimaging, for the initial classification of brain injury in the near future (Papa et al., 2008). As the hypoxic injury pattern on MRI is not diagnostic for 7–14 days, a blood biomarker that could fill the clinical gap for predicting current and worsening neurological status or long-term disability and would have great clinical utility. With the combinations of different pathophysiology related to each biomarker, a multi-biomarker analysis would seem to be the most effective way to assess brain injury and would likely increase diagnostic accuracy (Yokobori et al., 2013).

Blood biomarkers offer an objective and quantitative way to identify and follow a neonate with brain injury. They could be used to triage neonates to the current standard of 72 h of hypothermia as well as investigational therapies such as erythropoietin, xenon gas, melatonin and various forms of stem cell treatment. Blood biomarkers could possibly provide information about the extent of injury in the acute phase before ultrasound or MRI can identify abnormalities and determine the timing of injury as well. Blood biomarkers measured noninvasively using near-infrared spectroscopy (Torricelli et al., 2014) or optoacoustic techniques (Petrov et al., 2012a,b) may also allow quantitative monitoring of the fetal brain for injury during the intrapartum period through a dilated cervix with ruptured membranes via a probe attached to the fetal head similar to the currently used fetal scalp clip for measuring heart rate. Rather than imprecise measures of fetal and neonatal brain injury such as fetal heart rate abnormalities, meconium, cord gas at delivery, and Apgar scores, a multiplex combining glial and neuronal biomarkers could provide objective evidence of the extent and pattern of injury which would lead to improved identification of the brain injured fetus or neonate, operative delivery only in the presence of a significant risk of HIE, triage to appropriate therapy after birth and serve as an objective measure of the effectiveness of treatment for the brain injured baby.

## Author contributions

All authors listed, have made substantial, direct, and intellectual contribution to the work, and approved it for publication.

## Funding

Ernest Graham and Frances Northington are supported by the Cerebral Palsy International Research Foundation. Frances Northington is supported by R01 HD070996 and R01 HD074593. Irina Burd is supported by NICHD K08 HD073315-01. Allen Everett is supported by NHLBI R01 HL119664 and NIBIB R21EB018426

### Conflict of interest statement

The authors declare that the research was conducted in the absence of any commercial or financial relationships that could be construed as a potential conflict of interest. The reviewer MO and handling Editor declared their shared affiliation, and the handling Editor states that the process nevertheless met the standards of a fair and objective review.
